# Association between HLA genotypes and COVID-19 susceptibility, severity and progression: a comprehensive review of the literature

**DOI:** 10.1186/s40001-021-00563-1

**Published:** 2021-08-03

**Authors:** Filippo Migliorini, Ernesto Torsiello, Filippo Spiezia, Francesco Oliva, Markus Tingart, Nicola Maffulli

**Affiliations:** 1 Department of Orthopaedic and Trauma Surgery, RWTH University Hospital, Pauwelsstraße 30, 52074 Aachen, Germany; 2 Department of Medicine, Surgery and Dentistry, University of Salerno, Via S. Allende, 84081 Baronissi, SA Italy; 3 Ospedale San Carlo Potenza, Via Potito Petrone, 85100 Potenza, Italy; 4 Faculty of Medicine, School of Pharmacy and Bioengineering, Keele University, Thornburrow Drive, Stoke on Trent, England; 5 Barts and the London School of Medicine and Dentistry, Centre for Sports and Exercise Medicine, Mile End Hospital, Queen Mary University of London, 275 Bancroft Road, London, E1 4DG England

**Keywords:** COVID-19, SARS-CoV-2, MHC, HLA, Genotypes, Haplotypes, Polymorphisms

## Abstract

The COVID-19 pandemic has markedly impacted on cultural, political, and economic structures all over the world. Several aspects of its pathogenesis and related clinical consequences have not yet been elucidated. Infection rates, as well morbidity and mortality differed within countries. It is intriguing for scientists to understand how patient genetics may influence the outcome of the condition, to clarify which aspects could be related the clinical variability of SARS-CoV-2 disease. We reviewed the studies exploring the role of human leukocyte antigens (HLA) genotypes on individual responses to SARS-CoV-2 infection and/or progression, discussing also the contribution of the immunological patterns MHC-related. In March 2021, the main online databases were accessed. All the articles that investigated the possible association between the HLA genotypes and related polymorphisms with susceptibility, severity and progression of COVID-19 were considered. Although both genetic and environmental factors are certainly expected to influence the susceptibility to or protection of individuals, the HLA and related polymorphisms can influence susceptibility, progression and severity of SARS-CoV-2 infection. The crucial role played by HLA molecules in the immune response, especially through pathogen-derived peptide presentation, and the huge molecular variability of HLA alleles in the human populations could be responsible for the different rates of infection and the different patients following COVID-19 infection.

## Background

In December 2019 a cluster of pneumonia cases caused by an unknown etiological agent broke out in Wuhan [1]. A novel coronavirus was subsequently identified, sequenced and finally confirmed as the agent that caused the disease [2, 3]. In February 2020 the pathogen was named “Severe Acute Respiratory Syndrome Coronavirus-2” (SARS-CoV-2) by the International Classification Committee of Viruses [4]. At the same time, the associated disease was named “Coronavirus Disease 2019” (COVID-19) by the World Health Organization (WHO). COVID-19 currently represents a global pandemic with over 140 million confirmed cases and nearly 3 million deaths worldwide (updated to April 2021) [5]. One year after its appearance the infection has spread on all continents and the emergence of SARS-CoV-2 has caused one of the major public health and economic crises [6]. Considering the current situation, it would be useful to identify diagnostic and therapeutic targets, as well reliable predictors, to counter this pandemic. In this context, the role of the Human Leukocyte Antigens (HLA) complex appears particularly interesting, since its genetic variability is directly associated with individual variations in the immune response against pathogens and susceptibility to infectious diseases [7].

### Coronavirus

Coronaviruses are a wide group of viruses of enveloped positive-sense single-stranded RNA [8, 9]. Various types have been described: alpha, beta, delta, and gamma, but only alpha and beta coronavirus are known to infect humans [10, 11]. SARS-CoV-2 is a Betacoronavirus that shows many similarities [12, 13], including genomic [14, 15] and immune system response [16–20], to other coronaviruses [21], especially SARS-CoV and MERS-CoV [22–25]. Given the genomic similarity of SARS-CoV-2 to coronaviruses isolated in bats, the virus likely has a zoonotic origin, with bats themselves a natural reservoirs and other animals (e.g., snakes) as intermediate hosts [26, 27]. However, currently the predominant mode of transmission is between humans, through droplets emitted with coughs and sneezes [28]. The virions structure is characterized by the nucleocapsid protein and the spike glycoprotein. The glycoprotein S determines the specificity of the virus for the epithelial cells of the gastrointestinal and respiratory tracts because it is able to interact with transmembrane serine protease 2 (TMPRSS2) and angiotensin converting enzyme 2 (ACE2) receptors [29, 30]. ACE2 receptors are ubiquitous and widely expressed, in particularly on the cell surface of type II pneumocytes [31, 32]. The virus, therefore, shows high tropism for the lungs, causing fever, fatigue, cough, dyspnea, and pneumonia (CAP) [33]. Most patients present mild disease and develop an efficient immune reaction [34]. Some patients develop acute respiratory distress syndrome (ARDS), requiring intubation and mechanical ventilation (MV) in and intensive cares. Patients, often, suffer multi-organ dysfunction with high risk of death [34]. Patients over 65, particularly males, with comorbidities or organ-associated pathologies are at a higher risk of developing a severe, critical or even fatal disease course [35]. Type I diabetes mellitus has been also associated with severe clinical syndrome of SARS-CoV-2 infection. Hypertension and cardiovascular disease, obesity and/or a pro-inflammatory, and pro-coagulative state are also associated to the risk of worse outcome [36]. A dysregulated immune response participates in the sudden deterioration of COVID-19 patients, causing damage to infected and uninfected tissues. As with SARS-CoV-1 [37] and MERS-CoV [38, 39], children showed low susceptibility to the disease [40, 41]. Infection rates seem similar to those seen in adults [42], but only 5.9% of pediatric cases were severe or critical, possibly consequent to the lower binding ability of the ACE2 receptor in children or generally higher levels of antiviral antibodies [43].

### HLA locus

The human leukocyte antigen (HLA) gene complex is a locus of genes present on chromosome 6 that encodes proteins known ad major histocompatibility complexes (MHC). They constitute a specific group of molecules expressed on the cell surface, crucial for the recognition of non-self-molecules by the acquired immune system [44]. The essential function of MHCs is to bind and expose antigens derived from pathogens, to present them to the appropriate T lymphocytes, triggering the immune response. In particular, MHC molecules class I expose peptide antigens, present within the cytoplasm activating CD4 T-cell response [45], while MHC molecules class II expose peptide antigens present in the extracellular space activating CD8 T-cell response [46]. As HLA genes are a critical component of the antigen presentation pathway, they play a vital role in determining susceptibility to infectious disease. The HLA alleles are variable and polymorphic, and individuals with different HLA genotypes may trigger different immune responses against pathogens [47, 48].

## Materials and methods

### Search strategy

The literature search of the present systematic review was conducted according this protocol:

- Patients: SARS-CoV-2 infection;
- Comparison: HLA alleles;
- Outcomes: susceptibility, severity and progression of COVID-19.

### Literature search

In April 2021 the following databases were accessed: Pubmed, Embase, Scopus, Web of Science, Google Scholar. The following keywords were used in combination: coronavirus, COVID-19, SARS-CoV-2, SARS-CoV-1, MERS, infection, susceptibility, severity, progression, HLA, MHC, haplotypes, genotypes, locus, genes, alleles, polymorphisms, frequency, antigens, epitope, peptide, binder, factors, outcome, affinity, T cells, B cells, NK cells, lymphocyte, antibodies, association, correlation, genetic, pathogenesis, disease, immunology, virus, system, humoral, cellular, response, adaptive, interaction, epidemic, role, manifestation, clearance, risk, cytokines, dysregulation, affinity, clinic, injury, prognosis, diagnosis, therapy, variability, distribution, inflammatory, reaction, mortality, morbidity. If title and abstract matched the topic, the full-text was accessed. The bibliographies of the full-text articles were also screened for inclusion. Disagreements were solved by a third author (**). All the articles that investigate possible association between the HLA genotypes and related polymorphisms with susceptibility, severity, and progression of COVID-19 were considered. According to the authors language capabilities, articles in English, French, German, Italian, and Spanish were considered.

## Results

Based on the experience gained during the previous severe acute respiratory syndrome (SARS) and middle east respiratory syndrome (MERS) epidemics, it is likely that both innate and adaptive host immunity play a role in viral clearance, disease severity and the different clinical manifestations of the disease [49–52]. Studies on the SARS-CoV-1 virus identified HLA polymorphisms associated with the disease risk in the Asian population [53–58]. Also, in SARS-CoV-2 infection, different HLA alleles of the major histocompatibility complex may define individual susceptibility to infection [59].

### Immune system

Recent data suggest that appropriate innate and adaptive T cell-mediated humoral and cellular immune responses could help elimination of the SARS-CoV-2 virus, which in most cases coincides with clinical recovery [60]. On the other hand, an excessive cell-mediated and dysregulated innate and adaptive immune response can lead to an aggressive inflammatory reaction with the release of large amounts of pro-inflammatory cytokines. This condition, known as “cytokine storm”—resulting from the excessive production of cytokines by immune cells such as the innate dendritic cells, macrophages, natural killer (NK) cells, and the adaptive T and B cells—directly correlates with lung injury, ARDS and MOF, and leads to an unfavorable prognosis [61, 62]. Immunogenetic variation in humans could be an important target for clinical diagnosis and therapeutic intervention [63]. Binding between peptide epitopes and HLA proteins significantly contributes to cellular immune response mechanisms in human beings [64].

### Non-European HLA alleles

Several studies described the pivotal role of peptides in the specificity, magnitude and quality of both humoral and cellular immune responses. In silico studies have greatly facilitated analysis of the binding affinity between all the viral peptides of SARS-CoV-2 and different HLA class I genotypes. The HLA-B*46:01 allele has a low binding affinity, suggesting that subjects with this allele may have a higher risk of developing the more severe forms of COVID-19 [65], as previously shown with SARS-CoV [53]. On the other hand, the HLA-B*15:03 allele is reported to have the highest binding affinity for viral peptides [65]. On the contrary, the association with HLA-B*46:01 was not observed in the study by Yung et al., possibly because the T cell-mediated response could be a variable process, involving various factors, and not limited to HLA–peptide interactions [66]. However, the HLA-B22 serotype is a potential risk marker for SARS-CoV-2 infection [67]. In this regard, a recent study about the HLA binders reported that five B22 alleles (B*54:01, B*55:01, B*55:07, B*55:12 and B*56:01) were among the 94 weakest HLA-B binders to SARS-CoV-2, further suggesting B22 as a susceptibility marker [68]. The published data seem to suggest also a possible role of the HLA-B27 serotype in modulating SARS-CoV-2 infection [67]. B27 serotype mediates protection against HCV and HIV [69]. The same HLA markers may be also associated with susceptibility/resistance to all SARS-CoV-2, HIV, and HCV, may have a common general immune mechanism against viral infections. Indeed, all the three viruses are RNA viruses. In particular, SARS-CoV-2 and HCV are positive-sense RNA viruses, which share striking sequence and structural homology in their RNA polymerase and protease, central components for viral replication [70, 71]. Liver diseases have also been reported in COVID-19 patients, more prevalently in severe cases [72]. On the other hand, lymphopenia is the clinical hallmark of HIV infection, associated with increased COVID-19 severity [73]. Altered immune homeostasis could play a role in the pathogenesis of coronavirus disease. High binding affinity between SARS-CoV-2 epitopes and the HLA-A*02:06, HLA-B*52:01, and HLA-C*12:02 alleles has been also reported. In particular, two epitopes displayed strong binding affinity for HLA-A*24:02, HLA-A*02:01, and HLA-A*02:06 [74]. Unfortunately, these studies present mathematical predictions that need to be more statistically robust and better define the appropriate immunogenetic characteristics. Equally interesting is the study that reported the distribution of HLA allele frequencies in 82 Chinese individuals with COVID-19 and identified HLA-C*07:29 and HLA-B*15:27 and HLA-B as statistically significant [75]. However, in this study HLA-C*07:29 was found in one COVID-19 patient only, but in no individuals in the control group. Therefore, the significance of these findings should be interpreted with caution, and this result needs to be confirmed in studies with larger sample sizes. Another study of Chinese patients with COVID-19 reported that the HLA-A*11:01, HLA-B*51:01 and HLA-C*14:02 alleles were significantly associated with severe disease or worse outcome [76]. In another in silico analysis, the association of HLA gene polymorphisms with prevalence and mortality of COVID-19 was examined and a possible association between HLA-A*02:01 and an increased risk for infection were identified. This allele had a relatively lower ability to present SARS-CoV-2 antigens compared with other frequent HLA class I molecules, HLA-A*11:01 and HLA-A*24:02 [77]. The HLA-A*24:02 allele was also involved in a study on bronchoalveolar lavage fluid and blood samples of COVID-19 patients [78], while the HLA-A*11:01 allele, together with the HLA-A*02:06 and HLA-B*54:01 alleles, could protect against infection [79].

### European HLA alleles

In Italy, a study investigated whether specific class I HLA alleles could explain the huge differences observed for the spread of SARS-CoV-2 infection between Northern and Southern Italy. They compared HLA allele prevalence retrieved through the Italian Bone-Marrow Donor Registry with the incidence of SARS-CoV-2 infections in the different geographical regions. They showed how HLA-A*25, B*08, B*44, B*15:01, B*51, C*01, and C*03 was positively associated with the incidence of SARS-CoV-2 infection, while HLA-B*14, B*18, and B*49 showed an inverse association. After applying a multiple regression model to eliminate confounding factors, only the HLA-C*01 and HLA-B*44 alleles, which are present with a higher frequency in the northern regions of Italy, remained positively associated with COVID-19. In addition, this was confirmed by a sub-analysis between different provinces of the same region [80]. This epidemiological analysis has made it possible to identify specific class I HLA alleles that are potentially unable to present a sufficient amount of virus-derived epitope peptides and, consequently, to trigger an adequate immune response to counteract SARS-CoV-2 infection. In this context, two individuals carrying the same antigen but different HLA profile may give rise to a completely different T cell-mediated immune response, since they may have completely different amounts of HLA-specific antigen-derived epitopes. This hypothesis has been confirmed in several studies concerning a number of different viruses as well as tumor antigens and autoimmune models [81–87]. It can be assumed that, in these patients, the virus may freely spread from the oropharyngeal mucosae, starting a more efficient replication. Consistently, both HLA-B*44 and C*01 alleles, identified as possibly permissive to SARS-CoV-2 infection in Italy, have also been associated to known inflammatory autoimmune diseases [88–92], a fact that highlights their ability to trigger non-proficient and often inappropriate immunological reactions. Interestingly, the inheritance of HLA-B*44 underlies susceptibility to recurrent sinopulmonary infection [93]. A further consideration stems from the knowledge that the HLA-C*01 allele, which was the most permissive to SARS-CoV-2 infection in that study, also represents the specific ligand of killer cell immunoglobulin like receptors (KIRs), KIR2DL2 and KIR2DL3 [94–96]. These receptors are able to inhibit the activity of natural killer cells, which represent the first line of host defense to the infection before the occurrence of a more specific T cell response [97]. A recent report found that peptides bound to HLA-C*05:01 are recognized by one of the activating KIR (KIR2DS4) [98] and this allele was significantly associated with the risk of death from COVID-19 [99]. Again, in Italy, another study, performed in Sardinia, evaluated statistically significant association between local haplotypes and COVID-19. Almost a sanctuary throughout the human history, Sardinia could represent an ideal place to detect immunogenic factors potentially involved in resistance or susceptibility to SARS-CoV-2 infection. Overall, the analysis of the HLA alleles and haplotype frequencies showed 7 HLA alleles or haplotypes with a protective effect against SARS-CoV-2 infection, and 5 alleles or haplotypes that were associated with an increased susceptibility to infection. The most interesting alleles, after correction for multiple comparisons, were HLA-A*23:01 and HLA-DRB1*08:01. These two alleles were exclusively present in patients with a moderate or severe disease course. However, HLA-A*23:01 is an uncommon allele in the Sardinian population, and it is therefore difficult to assess its effect on the evolution of the disease. Only the HLA-A*30:02, B*14:02, C*08:02 three-loci haplotype maintained statistically significance relation after correction of the P values. This haplotype strongly correlated with disease severity [100]. Again in Italy, another study identified a significant association with a higher susceptibility to the disease was found for HLA-DRB1*15:01, HLA-DQB1*06:02 and HLA-B*27:07 after applying the Bonferroni’s correction for multiple tests [101]. The increased frequencies observed for DRB1*15:01 and DQB1*06:02 in the 99 severe affected COVID-19 Italian patients were not in line with the results obtained in a larger survey [102], which did not show any association between HLA and COVID-19, but confirmed published data [103] identifying these two alleles among seven HLA susceptibility alleles. Another interesting Italian study, through a geographic epidemiological analysis, observed that there are significant regional differences in the frequency of the two most common HLA haplotypes in the Italian population between the northern, central and southern regions, with HLA-A*01:01 g-B*08:01 g-C*07:01 g-DRB1*03:01 g (the most frequent haplotype nationwide) showing a decreasing frequency gradient, and HLA-A*02:01 g-B*18:01 g-C*07:01 g-DRB1*11:04 g (the second most frequent haplotype) an increasing frequency gradient from North to South. The geographical distribution of these haplotypes overlaps with that of COVID-19 in Italy, being linearly and significantly correlated in a positive/direct way (suggestive of susceptibility) for the haplotype 1 and in a negative/inverse way (suggestive of protection) for the haplotype 2, both for incidence and mortality [104]. In Spain, a study conducted in the ICUs of 6 hospitals in the Canary Islands found a trend to a higher infection rate of the alleles HLA-A*32, HLA-B*39 and HLA-C*16, but these *p* values were not significant after correction for multiple comparisons. Conversely, logistic regression analysis showed that the presence of the alleles HLA-A*11, HLA-C*01, HLA-DQB1*04 were associated with higher mortality after controlling for SOFA or APACHE-II [105]. A preprint article that considers the increased risk of hospitalization as parameter suggests that HLA-A*11:01, HLA-DQA1*01:02 and HLA-C*04:01 alleles are associated with greater severity disease. This is especially evident for COVID-19 patients with HLA-C*04:01, in whom disease prognosis measured by mechanical ventilation-free days was statistically significant after Bonferroni’s correction and may hold potential clinical value [106]. Regarding the different allele frequencies examined in that study, it must be emphasized that they vary greatly between regions, so it is well possible that alleles identified as risk alleles in some association studies have no weight in other populations, given their low presence in such populace [107].

## Conclusions

The association between HLA and COVID-19 deserves investigations on larger patient cohorts. Only limited sets of HLA alleles have been studied. Another limitation of the studies could be that, at present, it is not possible to assess the relative importance of the HLA type in correlation to known disease-modifying risk factors such as age and clinical comorbidities [108–111]. Meanwhile, these findings may provide new insights on pathogenesis of SARS-CoV-2, the design of vaccination programs, and more effective infection control to optimize the treatment, to management of patients with the disease, to identify those at greatest risk, and to reduce morbidity and mortality. Identification of high-risk subjects susceptible to SARS-CoV-2 infection could help to prevent virus spreading, reducing public health burden and prioritizing preventive medicine. Current scientific evidence suggests to integrate HLA testing into clinical trials and combine HLA typing with COVID-19 testing to more rapidly identify a predictor of viral severity in the population, and potentially adapt vaccination strategies to genotypically at risk populations.

## Data Availability

Not applicable.

## Abbreviations

COVID: Coronavirus disease
SARS: Severe acute respiratory syndrome
MERS: Middle east respiratory syndrome
HLA: Human leukocyte antigens
MHC: Major histocompatibility complex
S- and N-glycoprotein: Spike and nucleocapsid glycoprotein
TMPRSS2: Transmembrane serine protease 2
ACE2: Angiotensin converting enzyme 2
ARDS: Acute respiratory distress syndrome
MOF: Multiple organ failure
CAP: Community acquired pneumonia
ICU: Intensive care unit
MV: Mechanical ventilation
NK cells: Natural killer cells
CD: Cluster of differentiation
HCV: Hepatitis C virus
HIV: Human immunodeficiency virus
